# First draft genome sequence of a *Pectobacterium polaris* strain isolated in South Africa from potato tuber affected by soft rot

**DOI:** 10.1128/mra.00691-24

**Published:** 2024-09-09

**Authors:** Christiana C. Dapuliga, Maike Claussen, Stefan Schmidt

**Affiliations:** 1 Discipline of Microbiology, School of Life Sciences, University of KwaZulu-Natal, Pietermaritzburg, South Africa; The University of Arizona, Tucson, Arizona, USA

**Keywords:** *Pectobacterium polaris*, soft rot, potato, South Africa

## Abstract

A phytopathogenic strain of *Pectobacterium polaris* (designated SRB2) was isolated for the first time in South Africa from a potato tuber affected by soft rot. The draft genome of strain SRB2 encodes various plant cell wall-degrading enzymes and genes associated with biofilm formation and virulence. Antibiotic resistance genes were not detected.

## ANNOUNCEMENT

Members of the genus *Pectobacterium* (formerly *Erwinia*) are economically important phytopathogens secreting multiple plant cell wall-degrading enzymes (PCWDEs), such as pectate lyase, polygalacturonase, pectin esterase, proteases, and cellulases (1–4). In particular, enzymes breaking down pectin, a major structural component of plant cell walls (5), cause tissue maceration and vegetable deterioration (3, 6). *Pectobacterium polaris* is a recently described pectinolytic species of the genus *Pectobacterium* that was initially isolated in Norway from potato tubers exhibiting soft rot symptoms (7).

**Fig 1:**
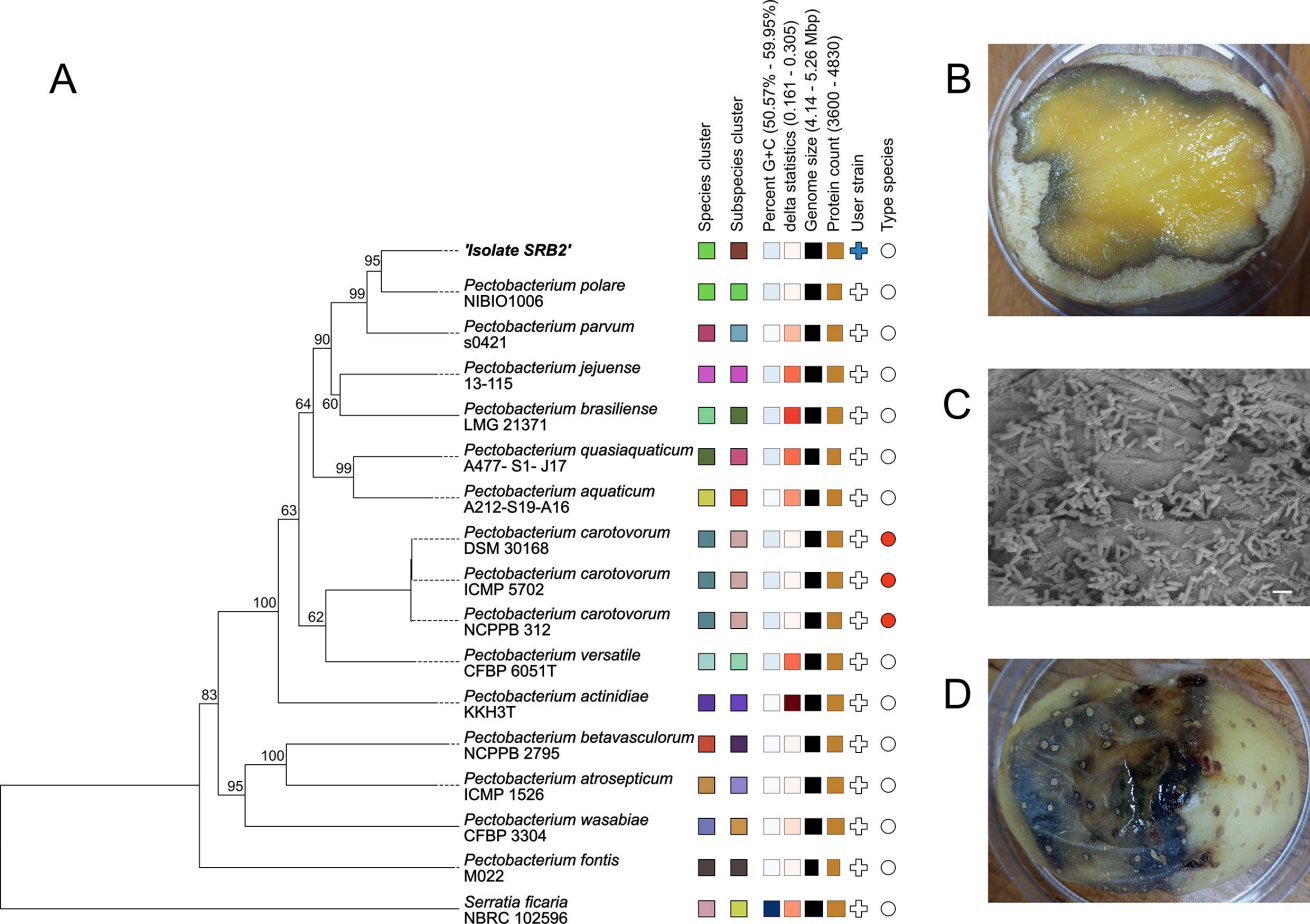
Phylogenomic comparison of *Pectobacterium polaris* isolate SBR2 with reference genomes and potato soft rot symptoms caused by the strain. (**A**) Midpoint-rooted phylogenetic tree inferred with FastME (2.1.6.1) from GBDP distances calculated from type strain genome sequences using the TYGS server. Branch lengths are scaled based on the GBDP distance formula d5; numbers shown are GBDP pseudo-bootstrap support values ≥60% (100 replications, average branch support = 79.4%). (**B**) Potato slice showing soft rot symptoms after inoculation with 10^5^ cells of strain SRB2 and subsequent incubation (24 h, 28°C). (**C**) Presence of a biofilm-like cell assembly of strain SRB2 on the surface of a matching inoculated potato slice (scale bar = 2 µm). (**D**) Potato tuber showing soft rot symptoms after inoculation with 10^5^ cells of strain SRB2 and subsequent incubation (72 h, 28°C).

*Pectobacterium polaris* strain SRB2 was isolated from a deteriorating potato tuber collected in October 2022 from a farm in the province of KwaZulu-Natal, South Africa. After surface disinfection with 70% ethanol, about 5 g of the decaying potato was homogenized in sterile saline (0.85%). A loop of the homogenized sample was streaked onto pectin-fortified MacConkey agar (22 g/L pectin added to MacConkey agar, pH 7.2), followed by 48 h of incubation at 28°C. Colonies exhibiting a clear halo due to pectin hydrolysis were selected, restreaked for confirmation, and purified on Mueller-Hinton agar. The pathogenicity of the chosen isolate, strain SRB2, was demonstrated by using 100-µL cell suspension (10^5^ cells) to infect healthy potato slices and tubers, with soft rot symptoms evident after 24 and 72 h of incubation at 28°C (Fig. 1), respectively. The isolate was stored at −20°C in brain heart infusion broth plus 20% glycerol. Strain SRB2, a gram-negative motile rod, is oxidase, urease, and indole negative, and β-galactosidase, protease, pyrrolidonyl-aminopeptidase, and γ-glutamyl-transpeptidase positive. Sanger sequencing (CAF, Stellenbosch) of the 16S rRNA gene amplified using primers fD1 + rP2 (8) identified strain SRB2 as *Pectobacterium polaris* (accession no. PP843237).

For shotgun sequencing, DNA was extracted using the Quick-DNA Miniprep Kit (Zymo, USA) from washed cells harvested from an overnight culture (Nutrient broth) by centrifugation, followed by DNA quantification using a Nanodrop 2000 spectrophotometer. A library was prepared using the FS DNA Library Prep Kit (NEB, USA), followed by sequencing (Illumina NextSeq) to produce 2 × 150 bp paired-end reads (Inqaba Biotech, South Africa). Employing the tools available in the BV-BRC genome analysis pipeline (9), raw reads were assembled (Unicycler V0.4.8, 500 bp minimum contig size) and polished (Pilon V1.23) after initial trimming and quality control (Trim Galore V0.6.5, Cutadapt V2.2), using default parameters (10–13). Annotation was done using PGAP (V6.7) (14), and the presence of antibiotic resistance genes, virulence genes, plasmids, and phages was analyzed using Resfinder (v4.5), PHI-base, Plasmidfinder, and Phastest (15–18).

The assembled genome (5,021,276 bp, 51.99% G + C) was 99.50% complete based on the benchmarking universal single-copy ortholog tool (BUSCO v5.0, gammproteobacteria_odb10) (19). Essential genomic features are summarized in Table 1.

**TABLE 1:** Genomic features and representative genes associated with phytopathogenicity of *Pectobacterium polaris* (*pro synon. P. polare*) strain SRB2

Feature	Description
Identity, source, date, location (country)	*Pectobacterium polaris* strain SRB2, rotting potato tuber, October 2022, KwaZulu-Natal (South Africa)
GenBank accession no.	JBEGDN000000000
Average short-read coverage	94
Largest contig, N50, and L50	828,269 bp, 357,547 bp, 5
Number of contigs	68
Genome length	5,021,276 bp
G + C content	51.99%
Protein-coding sequences, tRNA, ncRNA, rRNA	4,458, 67, 6, 3
Completeness (BUSCO)	99.50%
Antibiotic resistance genes and plasmids	Not detected
Intact prophages	3
rMLST profile best match	*Pectobacterium polaris*
Digital DNA:DNA hybridization (dDDH, d4) best type strain genome match	*Pectobacterium polaris* NIBIO1006^T^ (GCA_002307355.1) (74.90%)
Fast ANI best type strain genome match	*Pectobacterium polaris* NIBIO1006^T^ (GCA_002307355.1) (97.08%)
Representative genes encoding PCWDEs	*paeX/Y* (pectin acetylesterase, EC 3.1.1.6); *pelA/B/C/I/N/Z* (pectate lyase, EC 4.2.2.2); *pelW/X* (pectate disaccharide-lyase, EC 4.2.2.9); *pemA/B* (pectinesterase, EC 3.1.1.11); *ogl* (oligogalacturonate lyase, EC 4.2.2.6); *degP/Q/S, ftsH/htpX/pmbA/tldD, glpG, ptrA, sohB* (protease, EC 3.4.21.107/EC 3.4.24/EC 3.4.21.105/EC 3.4.24.55/EC 3.4.21); *bcsZ* (endoglucanase, EC 3.2.1.4)
Representative genes associated with biofilm formation, chemotaxis, virulence, and secretion	*bssS, bsmA*, *pilM/W* (biofilm regulation, biofilm formation); *cheA/R/W/Y/Z*, *qseB/C/G* (chemotaxis, quorum sensing); *ccdA, flgA*, *flhB*, *hlyD, lolA*, *metC/J*, *purD/H*, *pyrD*, *rsmA/B/S, sirB1, tadA*, *tolC, trkA/H, yefM* (virulence, pathogenicity); *gspB/C/D/E/F/G/H/I/J/K/L/S*, *fliP, hrpT, sctC/J/L/N/Q/R/S/T/U/V/W, virB9/B10, tssA/B/C/E/F/G/H/I/J/K/M* (secretion, T2/T3/T4/T6SS)

Using reference genomes, strain SRB2 was identified as *Pectobacterium polaris* by rMLST (20), establishing the average nucleotide identity (21) and dDDH (22). In addition, phylogenetic analysis using TYGS-LPSN (22) demonstrated that strain SRB2 is closely related to *P. polaris* (*pro synonymon P. polare*) NIBIO1006^T^ (Fig. 1; Table 1).

Our results confirm that genome analysis of locally isolated phytopathogens causing deterioration of staple foods such as potatoes is a valuable tool to identify and characterize isolates reliably.

## Data Availability

This Whole-Genome Shotgun project has been deposited at GenBank under the accession number JBEGDN000000000. The version described in this paper is JBEGDN000000000.1.The genome assembly was deposited under accession number GCF_040191255.1, under BioProject number PRJNA1117405. The raw reads were deposited under accession number SRR29203269.
